# *Annona cherimola* Miller Fruit as a Promising Candidate against Diabetic Complications: An In Vitro Study and Preliminary Clinical Results

**DOI:** 10.3390/foods9101350

**Published:** 2020-09-24

**Authors:** Marzia Vasarri, Emanuela Barletta, Santina Vinci, Matteo Ramazzotti, Andrea Francesconi, Francesco Manetti, Donatella Degl’Innocenti

**Affiliations:** 1 Department of Experimental and Clinical Biomedical Sciences, University of Florence, Viale Morgagni 50, 50134 Florence, Italy; marzia.vasarri@unifi.it (M.V.); emanuela.barletta@unifi.it (E.B.); vincisantina@gmail.com (S.V.); matteo.ramazzotti@unifi.it (M.R.); 2 Santa Maria Annunziata Hospital, Diabetology and Metabolic Diseases Unit, Via dell’Antella 58, Ponte a Niccheri, Bagno a Ripoli, 50012 Florence, Italy; a.francesconi13@gmail.com (A.F.); francesco1.manetti@uslcentro.toscana.it (F.M.)

**Keywords:** *Annona cherimola* Miller, anti-glycation, advanced glycation end products (AGEs), α-glucosidase, diabetes, diabetic complications, functional food

## Abstract

Diabetes is a chronic metabolic disease with a strong social impact worldwide. Under chronic hyperglycemia, protein glycation strongly contributes to diabetes-related complications onset. Anti-glycation agents and inhibitors of α-glucosidase are often therapeutically used to control postprandial glycemia in order to prevent development of long-term diabetic complications. Given drug resistance and adverse effects of conventional antidiabetic therapies, the discovery of new effective and non-toxic naturally occurring compounds is needed to prevent and/or to manage life-threatening diabetic complications. *Annona cherimola* Miller fruit has been used in Mexican traditional medicine as natural remedy against diabetes. In this work, the in vitro anti-glycation and anti-α-glucosidase roles of *Annona cherimola* Miller pulp extract (CE) were investigated. Moreover, healthy and diabetic subjects were enrolled in a cross-over design intervention study aimed at investigating the effects of pulp intake on postprandial glycemia. This work shows that CE was able to inhibit albumin glycation in vitro and to inhibit α-glucosidase enzyme. Furthermore, the pulp intake did not contribute to an increase in postprandial glycemia, making it a suitable source of health-promoting phytonutrients and a potential functional food in diabetics and pre-diabetics diet.

## 1. Introduction

Diabetes mellitus is a heterogeneous metabolic disease characterized by an imbalance of glucose homeostasis and consequent disturbances in fats and proteins metabolism [1]. According to the World Health Organization (WHO, 2020), all over the world, prevalence of diabetes in the adult population doubled in 2014 compared to 1980 (from 4.7% to 8.5%). These data reflect the increase over the past few decades in associated risk factors, including overweight and obesity [2]. 

In diabetic patients, the hyperglycemic condition in the absence of treatment is closely related to an increased risk of other serious health problems. Keeping the glycemic index in the correct range is the primary goal of healthcare organizations in diabetic patients [3]. An inevitable consequence of a long-lasting hyperglycemic state is an increased accumulation of advanced glycation end products (AGEs), a complex and diversified group of products of the non-enzymatic Maillard reaction between reducing sugars and macromolecules. Non-enzymatic glycation process can involve both plasma and basement membrane proteins, altering their molecular conformation, interrupting the enzymatic activity, or interfering with receptor function [4]. Among several plasma proteins, human serum albumin (HSA) is the most used for studying AGEs formation in vitro [5]. HSA, a 66.4 kDa (pI = 4.7) protein very susceptible to non-enzymatic glycation due to its high concentration (about 35–50 g/L) and to its half-life of about 20 days [6], undergoes structural and functional changes by binding reducing sugars. 

Although protein glycation products generally arise from normal metabolism, they are excessively accumulated in circulation and tissues in diabetic patients, where they can cause deleterious and often irreversible cellular damage. Indeed, it is known that AGEs formation and accumulation can contribute to the onset of diabetic complications, including nephropathy, retinopathy, cardiomyopathy, neuropathy, and other chronic diseases as rheumatoid arthritis, osteoporosis, and aging [7]. The ability of AGEs to promote oxidative stress and inflammation, either by binding cell membrane receptors or by cross-linking with proteins, is among the main causes of the damaging effects of AGEs [8]. 

In this regard, the search for effective AGEs inhibitors is undoubtedly a valid strategy to prevent or delay the formation and the accumulation of AGEs and therefore to limit the risk of developing diabetic complications. Over the past three decades, several inhibitors of protein glycation have been identified. Aminoguanidine was the first synthetic inhibitor discovered in 1986; despite being an outstanding protein glycation inhibitor, it has never been approved for clinical use, mainly because of its cellular toxicity. Other drugs already approved by the Food and Drug Administration (FDA) (USA) (such as aspirin, metformin, diclofenac, etc.) are not effective enough to inhibit the glycation process under chronic hyperglycemia conditions [9]. Since the number of anti-glycation agents is limited, there is an urgent demand for new and non-toxic compounds. 

Moreover, since α-glucosidase enzyme is one of the main glycolytic enzymes responsible for digesting carbohydrates in the gastrointestinal tract, α-glucosidase inhibitors are often used as a therapeutic strategy to monitor glucose in diabetics by reducing the intestinal carbohydrate metabolism rate and achieving better glycemic control [10]. Increasing evidence reveals the presence of α-glucosidase inhibitors in numerous plants, thus motivating researchers to explore several diverse plants as a source of biologically active compounds with hypoglycemic activity [11,12,13]. Although antidiabetic drugs have been improved over the past decade, drug resistance is still a major concern to be addressed with effective approaches. In this regard, there is a growing scientific interest in identifying new effective natural anti-glycation agents and inhibitors of α-glucosidase enzymatic activity.

For many years, medicinal plants have been traditionally used by different cultures around the world to manage diabetes and related complications. Over time, research on herbal medicines has gained importance in the search for new effective and safe antidiabetic therapeutic agents [14]. 

Today, herbal medicines and functional foods capable of modulating physiological effects are the target of renewed scientific interest for the management of diabetes and related complications [15,16]. 

Nowadays, several studies show that a healthy diet can prevent the development of certain diseases [17]. The daily intake of large quantities of fruit and vegetables, in addition to caloric restriction and regular exercise, is the basis of a healthy lifestyle and prevention of various chronic health problems. The high intake of phytonutrients (in particular, vitamins and plant phenols) can contribute to the reduction of the risk of various metabolic pathologies, including diabetes and related complications [18,19]. In this regard, there is a rich literature disclosing the benefits derived from the dietary consumption of tropical or subtropical fruits (such as mango, papaya, guava, and persimmon) [20,21]. 

*Annona cherimola* Miller, belonging to the Annonaceae family and vulgarly called “cherimoya”, is a tropical fruit that is found predominantly in northern Peru, southern Ecuador, and other subtropical areas but also in tropical or subtropical regions of America, Asia, Africa, and southern Europe [22]. 

In Italy, the cultivation of *Annona cherimola* Miller is limited in the Reggio Calabria area (Italy), given the specific pedoclimatic conditions [23], where it has been given the denomination of municipal origin (Denominazione comunale di origine—De.C.O.).

Anthropological studies suggest that *Annona cherimola* Miller fruit was a basic food of the Incan diet [22]. In the ethnopharmacological perspective, *Annona cherimola* Miller has been widely used in Mexican traditional medicine as a natural remedy for treatment of various ailments, including cough, fever, and headache as well as gastrointestinal and skin diseases [24]. 

The Mexican tradition has also handed down the use of *Annona cherimola* Miller leaves as a natural remedy for diabetes thanks to its hypoglycemic properties [25]. In vivo studies in alloxan-induced diabetic rats support the use of *Annona cherimola* leaves extracts as an anti-hyperglycemic agent [26]. Furthermore, literature indicates that extracts of *Annona cherimola* Miller leaves have antihypercholesterolemic [27], antihyperlipidemic [28], antidepressant [29], and anti-secretory [30] properties. Even the peel and the pulp of *Annona cherimola* Miller have shown potential health benefits due to their antioxidant, radical scavenging, and metal chelating properties, attributable to their high phenolic compounds content [31,32,33]. 

In the context of ongoing research on new therapeutic approaches against diabetes and associated complications, the present study was carried out to investigate the in vitro anti-glycation and anti-α-glucosidase effects of a hydroalcoholic extract from *Annona cherimola* Miller fresh pulp. Furthermore, the effect of *Annona cherimola* Miller pulp fruit on changes in postprandial blood glucose was investigated both in healthy individuals with normoglycemia and in type 2 diabetes mellitus patients (DM2) through a cross-over design intervention study.

## 2. Materials and Methods 

### 2.1. Materials and Reagents

Human serum albumin (HSA), D-glucose (Glc), aminoguanidine (AG), α,α-Diphenyl-β-picrylhydrazyl (DPPH), 3-(2-Pyridyl)-5,6-diphenyl-1,2,4-triazine-4′,4″-disulfonic acid sodium salt (Ferrozine™), Folin–Ciocalteau’s reagent, gallic acid, ascorbic acid, α-glucosidase enzyme (EC 3.2.1.20), p-nitrophenyl-α-D-glucopyranoside (pNPG), and all other chemicals and solvents were purchased from Sigma Aldrich-Merck (Saint Louis, MO, USA). Bio-Rad (Hercules, CA, USA) provided all electrophoresis reagents and Coomassie Brilliant Blue G, while disposable plastics were purchased from Sarstedt (Nümbrecht, Germany).

### 2.2. Preparation of Annona cherimola Miller Pulp Extract

The *Annona cherimola* Miller fruits were harvested in October by authorized staff of the Francesco Caridi Farm (Gallico, Reggio Calabria, Italy). To minimize the loss of vitamins, bioactive compounds, and other nutrients, the fruits of *Annona cherimola* Miller were transported at 10 °C in thermal containers. Fruits were removed manually from peel and seeds (non-edible portions), while pulp (edible portion) was cut into small pieces and homogenized. The *Annona cherimola* Miller extract was obtained by adding 60 g of the homogenized pulp sample to 140 mL of ethanol. After 12 h incubation at room temperature under continuous stirring, the mixture was centrifuged for 20 min at 7500× *g* for pelleting solid material, and the recovered supernatant was further centrifuged for 5 min at 14,000× *g*. The hydroalcoholic extract was dispensed in 1 mL aliquots and then dried using a Univapo vacuum concentrator (UniEquip, Planegg, Munchen, Germany). Dry extract (equivalent to 1 mL extract) of *Annona cherimola* Miller was dissolved in 500 µL of bi-distilled water before use and is hereinafter named CE (cherimola pulp extract). 

### 2.3. Determination of Total Content of Polyphenols and Carbohydrates 

Total phenolic (TP) and carbohydrate (TC) content of CE was assessed using the Folin–Ciocalteau’s and the phenol-sulfuric acid methods, respectively, described in [34,35,36]. Gallic acid (0.5 mg/mL) and D-glucose (1 mg/mL) were used as reference to determine TP and TC values, respectively. 

### 2.4. Evaluation of Antioxidant and Radical Scavenging Activities 

The CE antioxidant and the free-radical scavenging activities were established using the ferric reducing/antioxidant power (FRAP) and DPPH assays, respectively [34,35,36]. Ascorbic acid (0.1 mg/mL) was used as reference in the range of 0–4 μg to evaluate both activities.

### 2.5. In Vitro Non-Enzymatic Glycation of Human Serum Albumin (aAGE)

Glycation of albumin was performed in vitro following the previously described method [5] with some modifications. Briefly, a thermal incubation of HSA (1 mg/mL) and Glc (500 mM) was carried out in phosphate-buffered saline (PBS; 1.37 M NaCl, 27 mM KCl, 100 mM Na_2_HPO_4_, 18 mM KH_2_PO_4_; pH 7.4) at 60 °C or 37 °C for 24 h and 48 h under stirring conditions (300 rpm) (Thermo-Shaker TS-100, Biosan, Riga, Latvija). Glycated albumin is hereinafter referred to as aAGE (advanced glycation end products of albumin). The HSA solution incubated without Glc under the same conditions was used as a control. Formation of aAGEs was quantified by detecting the typical AGEs fluorescence intensity through a microplate spectrophotometer (Fluoroskan Ascent FL, Thermo Scientific™, Waltham, MA, USA), according to Sharma et al. [37] with some modifications. The excitation and the emission wavelengths were 396 nm (slit 5) and 485 nm (slit 5), respectively.

### 2.6. The In Vitro Inhibition of aAGE Formation

To determine the anti-glycation activity, the method of Vasarri et al. was executed with some modifications [5]. Briefly, HSA and Glc were thermally incubated in the presence of different amounts of CE. AG (5 mM), a known synthetic anti-glycation agent, was used as a positive control to inhibit the formation of aAGE. The inhibitory contribution of CE antioxidant activity to protein glycation was evaluated in the presence of ascorbic acid (ASC) equivalents. At 24, 48, and 72 h, 40 μL of trichloroacetic acid (TCA, 100% *w*/*v*) was added to 400 μL of aAGE solution to stop the in vitro glycation reaction. The TCA-stopped mixture was kept at 4 °C for 10 min and then centrifuged at 21,000× *g*. Precipitation with TCA was used to minimize interference for the fluorescence measurements. Thus, the precipitated material was resuspended in alkaline PBS (pH 10) and evaluated in the above-described fluorescence assay.

### 2.7. Native Polyacrylamide Gel Electrophoresis (N-PAGE)

The formation of aAGE was investigated by 12% N-PAGE under native condition (Tris-glycine buffer: 25 mM Tris and 192 mM Glycine, pH 8.3) as described by Vasarri et al. [5]. Samples were diluted with sample buffer (62.5 mM Tris-HCl pH 6.8, 25% (*w*/*v*) glycerol and 0.5% bromophenol blue) in a 1:1 ratio in the absence of reducing agents. Samples (1 μg) of glycated and non-glycated albumin were separated by N-PAGE for 100 min at 200 V, using Bio-Rad electrophoresis equipment (Mini-PROTEAN^®^). Protein bands were stained with Coomassie Brilliant Blue R-250 (1%) for one hour and then were destained overnight with a solution of 40% (*v*/*v*) methanol and 10% (*v*/*v*) acetic acid. Gel images were digitized using a commercial scanner.

### 2.8. α-Glucosidase Activity Assay

The enzymatic activity of α-glucosidase was determined according to the multiwell-adapted chromogenic method described by Moradi-Afrapoli et al. [38], with appropriate adjustments. Briefly, solutions of p-nitrophenyl-α-d-glucopyranoside (pNPG; 2.5 mM)—i.e., substrate solution—and α-glucosidase (0.5 unit/mL) from *Saccharomyces cerevisiae* yeast (EC 3.2.1.20)—i.e., enzyme solution—were solubilized in phosphate buffer (0.1 M, pH 6.9) in a final volume of 250 μL. Following a 10 min incubation at 25 °C, reaction was stopped by adding 50 μL of NaOH (125 mM). Absorbance was measured at 405 nm by using a microplate reader (iMARK, Bio-Rad, Philadelphia, PA, USA). The ability of CE to inhibit α-glucosidase activity was determined by performing this colorimetric assay at different concentrations of pNPG substrate (from 0.1 to 2.5 mM) with or without two different concentrations of CE—i.e., 7.5 and 15 μg/mL of dry extract. The inhibition mode of CE on α-glucosidase enzymatic activity was determined by analyzing the data with the Lineweaver–Burk double reciprocal plot, calculated according to Michaelis–Menten kinetics. Each data point was the result of at least three independent experiments. Kinetics data were processed with the R statistical software.

### 2.9. In Vivo Preliminary Clinical Study

#### 2.9.1. Enrollment of Participants 

Ten healthy normoglycemic volunteers and ten volunteer DM2 patients were enrolled for this preliminary clinical study. Healthy control subjects were recruited through direct contact with our staff. Patients with DM2 were recruited at the Santa Maria Annunziata Hospital (Florence, Italy) by screening the patient’s clinical data to identify their eligibility as DM2 participants in this preliminary clinical study. The following conditions were criteria for excluding patients from the study: food allergies, pregnancy, cardiovascular diseases, cancer, gastrointestinal diseases, or other chronic disease. Furthermore, all subjects enrolled in the study were non-smokers and not taking medications. All participants were of the same ethnicity (Italian) and completed a questionnaire covering demographic data including weight, height, and body mass index. Since this was a preliminary clinical study, participants were enrolled on a voluntary basis on the day of the test: 7 females and 3 males for the DM2 patient group and 8 females and 2 males for the healthy control group. The age of the enrolled subjects was also quite variable, a mean of 29 years for healthy subjects and a mean of 63 years for DM2 patients. The whole study was verified by an ethics committee of the Santa Maria Annunziata Hospital (Florence, Italy), and all participants gave their informed consent according to the laws in force.

#### 2.9.2. Cross-Over Design Intervention Study

Capillary blood was collected from all subjects by hospital healthcare personnel using a commercial finger-prick test (Breeze^®^ 2, Bayer). Blood glucose concentration of the analyzed subjects was determined from a drop (about 5 µL) of capillary blood. To determine the baseline blood glucose level, fasting capillary blood glucose was measured after overnight fasting of 8–10 h and before the meal intake (T0). For each subject, five capillary blood samples were collected both after a standard or a test meal. The standard meal consisted of a cappuccino and a brioche (Table 1).

The test meal consisted of standard meal supplemented with 100 g of fresh pulp of *Annona cherimola* Miller fruit (Table 2). 

Standard meal composition was elaborated and provided by the Diabetology staff of the Santa Maria Annunziata Hospital (Florence, Italy). Collection of additional capillary blood samples was performed in all subjects at 30, 60, 90, and 120 min after the meal.

#### 2.9.3. Clinical Testing Schedule

All participants visited the Diabetology Department of the Santa Maria Annunziata Hospital (Florence, Italy) between 06:00 and 08:00 after 8–10 h of overnight fasting. Capillary blood samples from this preliminary clinical study were part of the periodic blood glucose monitoring for DM2 patients. During this study, all the subjects were asked not to change their diet or physical activity and not to exercise when going to the clinic but to walk slowly or to be transported by a vehicle. Participants attended the clinic twice, once to test glycemic curve after consuming the standard meal and once to do the same after the test meal. All subjects were recommended to consume food in 10 min for both meals and then to sit quietly and refrain from eating or drinking during the 2 h trial period. Both groups started the sequence test on different days. The healthcare personnel employed for the collection and the measurement of blood glucose included registered nurses and doctors.

### 2.10. Statistical Analysis for In Vitro Experiments and Cross-Over Design Intervention Study

All values obtained from the in vitro experiments are expressed as the mean ± standard deviation of at least three experiments from three independent pulp extractions. The analysis of linear regression and the corresponding interpolations were executed with Excel (Microsoft Corporation, Redmond, WA, USA). Non-linear curve fitting was carried out with Origin 6.0 (OriginLab, OriginLab Corporation, Northampton, MA, USA). The clinical study data on the characteristics of the subjects were analyzed using descriptive statistical analyses with the R software. The independent T-test was used to examine differences in means for continuous normally distributed variables (age, height, weight, and body mass index), while the paired T-test was used to examine differences in means within groups. Incremental changes in the blood glucose concentrations of the analyzed subjects were calculated in relation to fasting in all postprandial periods. Incremental areas under the curve (AUC) for blood glucose values were determined between 0 and 120 min. Differences before and after the trial period were tested by paired T-test. Data following a non-normal distribution were examined through the Wilcoxon’s nonparametric test. Mean values were significantly different when *p*-value was <0.05. 

## 3. Results and Discussion

### 3.1. Biochemical Characterization of Annona cherimola Miller Pulp Extract

The hydroalcoholic extraction method from fruit pulps is widely used to recover a significant amount of hydrophilic compounds soluble enough to be easily assessed in biological media and widely representative of the properties of the main components of pulp or fruit juice and, above all, sufficiently stable to allow experiments in times when the fruit is not available on the market [40,41,42,43]. Given the high water content in the *Annona cherimola* Miller pulp (see Table 2), in this work, a hydroalcoholic extract was prepared from the pulp by adding only ethanolic solvent. 

Our hydroalcoholic extraction allowed us to recover about 4.8 ± 0.2 g of dry extract starting from 60 g of *Annona cherimola* Miller fresh pulp, and considering a standard resuspension volume, the concentration of analytes in each batch was 0.15 mg/mL. The edible portion of *Annona cherimola* Miller is known to be rich in water and poor in total protein and fat when compared to other fruits; it is especially known to be a good source of bioactive compounds as polyphenols [44,45]. Since there exists a close relationship between phenolic compounds and the beneficial properties of plant foods, scientific research is strongly interested in the determination and the characterization of the polyphenolic composition of fruits for their potential use in the manufacture of functional foods [46]. 

In this work, the total phenolic content (TP) of CE was determined by Folin–Ciocalteau assay. In particular, CE was found to contain 9.249 ± 0.41 mg of polyphenols (gallic acid equivalents) per gram of dry extract (Table 3). However, *Annona cherimola* Miller pulp has been proven to be highly carbohydrate-rich [45]. Here, the total carbohydrate content (TC) was assessed by phenol-sulfuric assay, highlighting a content of 3306 ± 154 mg of carbohydrates (equivalent to glucose) per gram of dry extract (Table 3). These data are extremely similar to those already discussed in the literature on the carbohydrate content in the *Annona cherimola* Miller pulp [31]. Since both polyphenols and carbohydrates are well recognized to act as scavengers for free radicals and, in general, as antioxidant agents, the antioxidant power and the radical scavenging activity of CE were investigated with FRAP and DPPH assays, respectively. Specifically, CE showed an antioxidant and radical scavenging activity of 7.212 ± 0.034 and 1.134 ± 0.082 mg (equivalents of ascorbic acid) per gram of dry extract, respectively (Table 1). 

These data support the great power of free radical scavenger of the extracts obtained from *Annona cherimola* Miller pulp, already reported in the literature [45]. In the context of diabetic pathology, oxidative stress plays an important role in the development of pathophysiological complications. To date, it is known that the integration of antioxidants in the daily diet is of fundamental importance in treatment and prevention of diabetic complications.

### 3.2. In Vitro Annona cherimola Miller Pulp Extract Anti-Glycation Activity

Diabetes and associated complications represent one of the most significant causes of morbidity and mortality in the world [47]. A state of chronic hyperglycemia often favors glycation of proteins and other biomolecules, leading to the onset of late diabetic complications. In this regard, protein glycation is both a marker of diabetic complications progression and the leading cause of diabetes-associated disorders [4]. Given the limited availability of effective anti-glycation agents and the current urgent need to manage diabetic complications, researchers are encouraged to identify novel non-toxic natural compounds capable of inhibiting protein glycation. 

**Figure 1:**
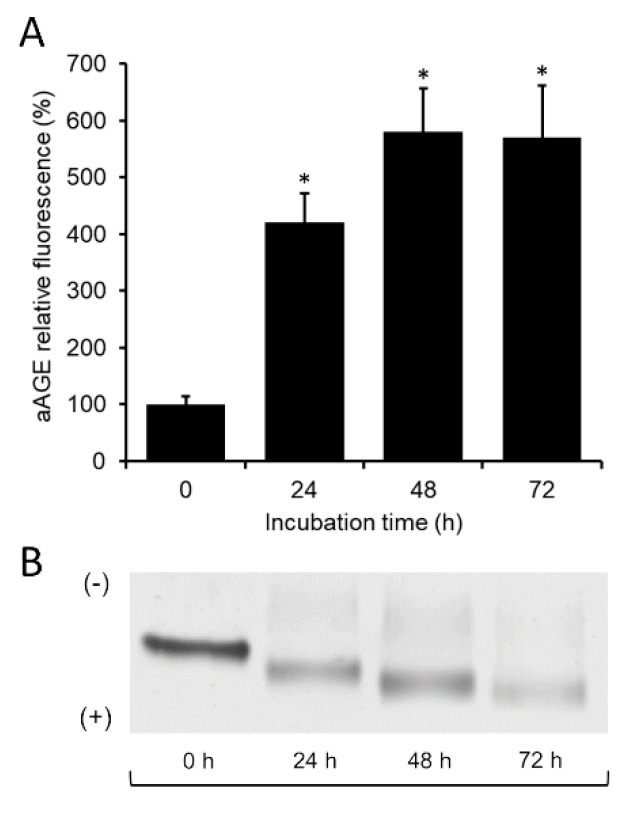
In vitro albumin glycation (aAGE). (**A**) Relative fluorescence intensity (λex/λem 396/485 nm) of aAGE obtained at different time points, ranging from 0 to 72 h. All values are reported as percentage ratio with respect to non-glycated albumin (aAGE at 0 h). Data are the average of at least three independent experiments. Error bars represent standard deviation. *: *p*-value < 0.05 vs. aAGE at 0 h. (**B**) Representative image of N-PAGE on aAGE electrophoretic migration obtained at different times. Symbols (+) and (-) represent the anode and the cathode, respectively, in gel N-PAGE.

Hence, taking into account the traditional anti-diabetic use of the *Annona cherimola* Miller leaves in Mexican traditional medicine as an agent with hypoglycemic properties [26], here, CE was investigated as a possible agent against HSA glycation. Specifically, albumin glycation was performed in vitro by incubating HSA (1 mg/mL) with Glc (500 mM) from 0 to 72 h at 60 °C. To verify albumin glycation, its intrinsic fluorescence intensity was measured. As shown in Figure 1A, the aAGE fluorescence intensity depended on the incubation time, showing an increase of approximately four times (420 ± 51.6%) and six times (580 ± 76.5%) at 24 h and 48 h, respectively, compared to non-glycated albumin (aAGE at 0 h). At 72 h incubation, aAGE fluorescence intensity remained almost unchanged (570 ± 90%) compared to 48 h. These findings strongly suggest that HSA underwent a conformation change due to glucose-induced glycation, supporting the successful production of aAGE. The glycation process compromises the albumin isoelectric point, causing the condensation of positive residues (arginine and lysine) with carbohydrates and thus decreasing the cationic charges of the glycation products [6]. Therefore, since the loss of positive charges favors the electrophoretic migration from the cathode to the anode, the formation of aAGE can also be proved by N-PAGE technique. As depicted in Figure 1B, the aAGE electrophoretic migration consistently increased from 24 h-formed aAGE to 72 h-formed ones, as expected by condensation of positive residues, as discussed above. This result is consistent with the incubation time-dependent rise in aAGE fluorescence intensity, suggesting that aAGE was widely produced at 72 h.

**Figure 2:**
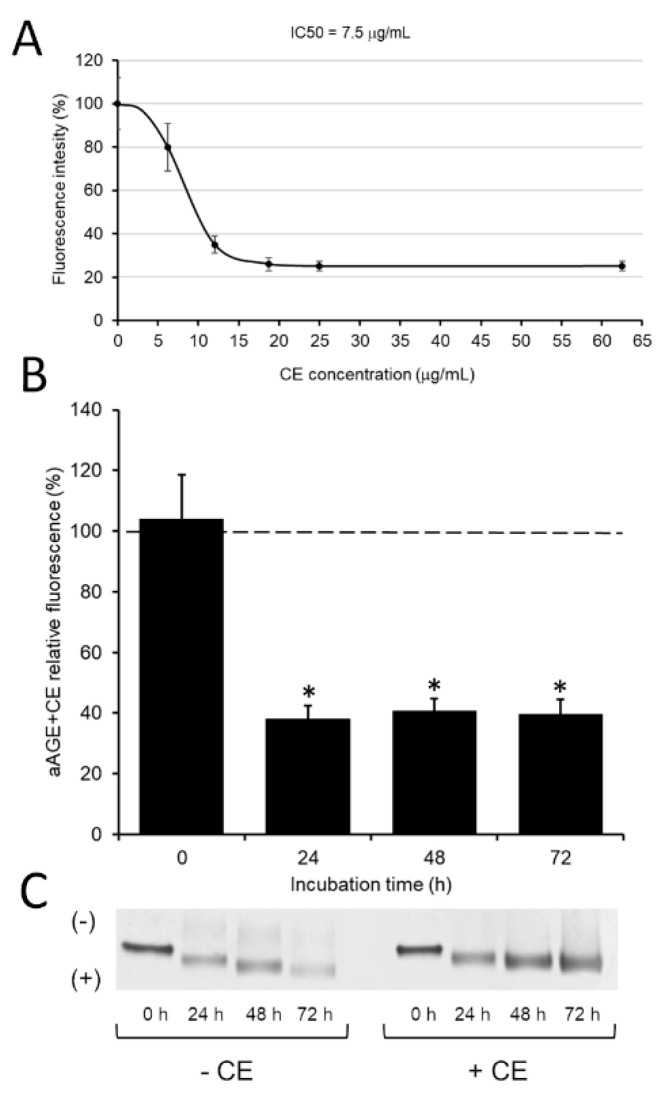
The inhibitory effect of CE on aAGE formation. (**A**) Evaluation of the concentration (μg/mL) required to reduce fluorescent aAGE formation by 50% (IC50 value). (**B**) Inhibition of the formation of fluorescent aAGE by CE (25 μg/mL) during albumin glycation over time. aAGE + CE relative fluorescence values are expressed as a percentage ratio with respect to those of aAGE formed over time in the absence of CE (represented by the dotted line). All values are the mean ± standard deviation of at least three independent experiments. Error bars represent standard deviation. *: *p*-value < 0.05 vs. aAGE formed in the absence of CE. (**C**) Representative image of N-PAGE on the electrophoretic migration of aAGE obtained at different times. Symbols (+) and (-) represent the anode and the cathode, respectively, in gel N-PAGE.

In order to investigate the inhibitory effect of CE on albumin glycation, the formation of aAGE was examined by incubating at 60 °C for 72 h HSA (1 mg/mL) and Glc (500 mM) in the presence of increasing doses of CE (from 0 to 62.5 μg/mL) and by monitoring the formation of fluorescent products at 485 nm emission wavelength. CE inhibited aAGE formation in a dose-dependent manner, as represented in Figure 2A. Specifically, CE exhibited the half maximal inhibitory concentration (IC50) value of about 7.5 μg/mL of dry extract and presented a maximum inhibition activity (approximately a 75% inhibition) at a concentration of 25 μg/mL. Then, CE anti-glycation role was monitored over time, from 0 to 72 h, incubating at 60 °C HSA (1 mg/mL) and Glc (500 mM) in the presence of CE. To ensure maximum inhibitory activity on albumin glycation, CE was added to glycation solution in a concentration of 25 μg/mL, corresponding to 2.5 mM of polyphenols (gallic acid equivalents) and an antioxidant activity equivalent to 0.3 mM ASC. As shown in Figure 2B, CE showed a clear ability to inhibit the aAGE production already at 24 h of incubation, causing a 60% reduction in the aAGE formation (38 ± 4.2% of aAGE fluorescence intensity) with respect to that of non-glycated albumin (aAGE at 0 h). However, CE maintained its anti-glycation activity high even in the subsequent 48 h and 72 h incubation times. By analyzing the N-PAGE electrophoretic migration of aAGE + CE samples obtained at the various time points, the anti-glycation role of CE was further supported by the slower electrophoretic mobility of aAGE + CE compared to that of aAGE for each time point (Figure 2C).

**Figure 3:**
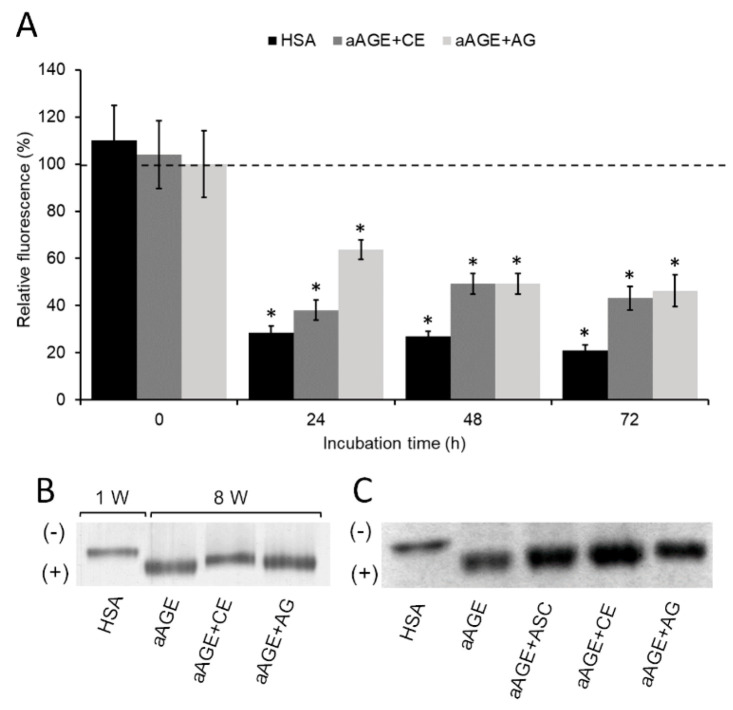
Anti-glycation role of CE. (**A**) Relative fluorescence intensity of aAGE obtained at different times in the presence of CE (25 μg/mL) or aminoguanidine (AG) (5 mM). Human serum albumin (HSA) solution without Glc was used as control and incubated under the same conditions. All values are expressed as a percentage ratio with respect to those of aAGE formed over time in the absence of any inhibitory agent (represented by the dotted line). All values are the mean ± standard deviation of at least three independent experiments. Error bars represent standard deviation. *: *p*-value < 0.05 vs. aAGE formed in the absence of any inhibitory agents. (**B**) Representative image of N-PAGE. Lane 1 shows the electrophoretic migration of HSA control incubated without Glc for 72 h at 60 °C, while the subsequent lanes show the variation of electrophoretic mobility between various aAGE samples obtained after 8 weeks of incubation at 37 °C. (**C**) N-PAGE representative image of the electrophoretic migration of aAGE samples obtained at 72 h incubation at 60 °C. Symbols (+) and (-) in Figure 3B,C represent the anode and the cathode, respectively, in gel N-PAGE.

As a control of inhibition, the glycation of HSA was carried out in the presence of AG (5 mM). As shown in Figure 3A, AG caused a reduction in the aAGE formation of approximately 35% (63 ± 4% of aAGE fluorescence intensity) at 24 h incubation and 50% at 48 h and 72 h incubation (50 ± 4% and 53 ± 7% of aAGE fluorescence intensity, respectively) with respect to that of non-glycated albumin (aAGE at 0 h). Interestingly, CE (25 μg/mL) was more effective than AG (5 mM) in inhibiting albumin glycation at 24 h incubation, while in subsequent incubation times, the activity of CE was comparable to that of AG. In order to evaluate the ability of CE to inhibit protein glycation in the most physiological conditions possible, the aAGE formation was verified by incubating HSA with Glc in the presence of the same CE concentrations for 8 weeks at 37 °C and evaluating the electrophoretic migration in N-PAGE. It was observed that aAGE + CE electrophoretic mobility was clearly reduced toward anode compared to that of aAGE + AG (Figure 3B). These results further confirm the strong inhibitory role of CE on albumin glycation. In order to rule out that the CE anti-glycation ability was exclusively due to its known antioxidant activity, the formation of albumin glycation products in the presence of ASC equivalents (0.3 mM) of CE was examined. A small difference in electrophoretic mobility between aAGE + CE and aAGE + ASC was observed in N-PAGE (Figure 3C), suggesting that the anti-glycation role of CE occurs somewhat independently of its antioxidant property. However, effective action against protein glycation process requires good antioxidant properties. Since the formation of glycation products is favored by oxidative reactions [48], antioxidant agents are an excellent tool for inhibiting this process [49]. Moreover, AGEs themselves can be a direct source of reactive oxygen species (ROS) and oxidative stress. The possibility of slowing down both the formation of AGEs and oxidative stress, given its well-known antioxidant properties [32], could make the *Annona cherimola* Miller pulp efficient in preventing the diabetic complications onset.

### 3.3. Annona cherimola Mill Pulp Extract Competitively Inhibits α-Glucosidase Enzymatic Activity

Currently, diabetic complications have turned to be more tedious and a serious problem to treat. In DM2 patients, α-glucosidase is one of the enzymes responsible for the digestion of carbohydrate. Hence, the inhibition of α-glucosidase enzymatic activity is one of the therapeutic goals in the management of diabetes, as it delays the carbohydrates absorption and digestion, thus alleviating postprandial hyperglycemia [50,51,52]. Various plant phytocompounds, including flavonoids, terpenoids, alkaloids, glycosides, anthocyanins, phenolic compounds, etc., have received much attention over the years for their diabetes management potential [13]. Some studies revealed the presence of α-glucosidase inhibitors in leaves, root, bark, and fruit extracts of various medicinal plants, such as in the leaves extract from *Morus alba*, mango (*Mangifera indica* L.), or in the extract of peel, seeds, pulp, and flakes of jackfruit (*Artocarpus heterophyllus*). Grape seed and green tea extracts have proven to be effective inhibitors of α-glucosidase activity [12]. 

**Figure 4:**
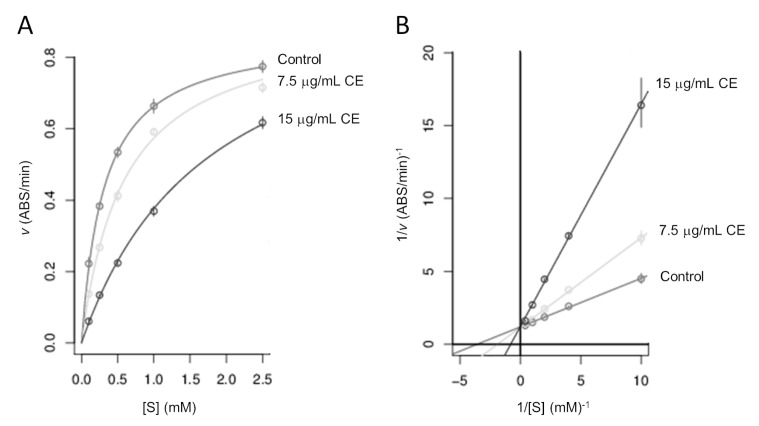
Inhibition of α-glucosidase enzymatic activity by *Annona cherimola* Miller pulp extract. Reaction conditions are reported in Materials and Methods section. (**A**) Michaelis–Menten kinetics plot and (**B**) Lineweaver–Burk plot generated on the Michaelis–Menten equation for inhibition of α-glucosidase at two different CE concentrations (μg/mL): 7.5 μg/mL and 15 μg/mL. In the plots, “v” stands for reaction velocity, and “[S]” stands for pNPG substrate concentration. The enzymatic reaction performed in the absence of CE was used as a control.

As discussed above, *Annona cherimola* Miller pulp extract has shown promising in vitro anti-glycation activity potentially useful in managing diabetes-related complications. To better understand whether CE has potentially useful bioactivity in the management of hyperglycemic conditions, in this work, the in vitro inhibitory action of CE on the α-glucosidase activity was evaluated. In particular, the mode inhibition of CE on α-glucosidase was investigated by analyzing Lineweaver–Burk plots through Michaelis–Menten kinetics. Various concentrations of pNPG substrate (from 0.1 to 2.5 mM) were used in the presence of two different CE concentrations: 7.5 μg/mL of dry extract—i.e., the concentration of CE inhibiting the in vitro formation of 50% aAGE—and a double concentration of 15 μg/mL of dry extract. From results obtained according to Michaelis–Menten kinetics study (Figure 4A), CE (Ki = 17.72 μg/mL) clearly inhibited α-glucosidase activity in a competitive and dose-dependent manner, as displayed by the Lineweaver–Burk plot in Figure 4B. In fact, by increasing the concentration of CE, Km raised, but Vmax remained the same as the common value in the *y*-axis, suggesting that CE functioned as a competitive inhibitor of α-glucosidase enzymatic activity. Using CE as a competitive inhibitor of this enzyme, it can be hypothesized that the consumption of *Annona cherimola* Miller pulp fruit or the pharmacological formulation of its extract could somehow contribute to slowing down sugars degradation and therefore to lowering the rise in postprandial glycemia in diabetics. 

Furthermore, given the expected future increase in the diabetic population worldwide, the search for new hypoglycemic agents from herbal medicines has become a great task for scientific research. Therefore, our results are in line with the search for new inhibitors on α-glucosidase as potential natural occurring candidates for antidiabetic therapies development.

### 3.4. Impact of Annona cherimola Miller Pulp Fruit on Changes in Postprandial Glycemia in Healthy Subjects and DM2 Patients

In patients with DM2, postprandial hyperglycemia is a considerable risk factor for disease progression and related disorders development. The postprandial phase is characterized by “hyperglycemic peaks”, which consist of abnormal and rapidly increased blood sugar level [53]. Patients with DM2 or pre-diabetic subjects must carefully monitor their fasting and post-meal blood glucose and follow a healthy and correct diet without taking foods with a high glycemic index. Since *Annona cherimola* Miller pulp extract showed, in this work, to have an in vitro anti-glycation property, it is reasonable to think of its potential use as a functional food in preventing diabetes-related pathophysiological conditions. However, it is necessary to note that the *Annona cherimola* Miller pulp intake contributes to raising postprandial blood sugar, both in diabetics and non-diabetics, thus increasing the risk of diabetic complications. 

In this regard, a cross-over intervention study was designed in this work to explore the effects of the ingestion of 100 g of *Annona cherimola* Miller edible portion on the postprandial glucose elevation, both in healthy subjects and in DM2 patients. Twenty subjects were contacted and invited to take part in the trial. Participants were distributed into two groups, one of ten healthy normoglycemic subjects (eight females and two males) with an average age of 29.3 ± 8.6 years, and one of ten DM2 patients (seven female and three male) with an average age of 63.90 ± 9.35. The baseline features of all participants are shown in Table 4. Given the difficulty in recruiting healthy subjects, the difference in gender, age, and BMI distribution was quite significant among healthy and diabetic individuals. Of the twenty participants, a healthy woman withdrew from the study for personal reasons (incomplete data of this subject were, however, included in the dataset), while nineteen subjects successfully completed the trial. Six healthy subjects did not participate in capillary blood sampling at 30 min after the standard meal.

**Figure 5:**
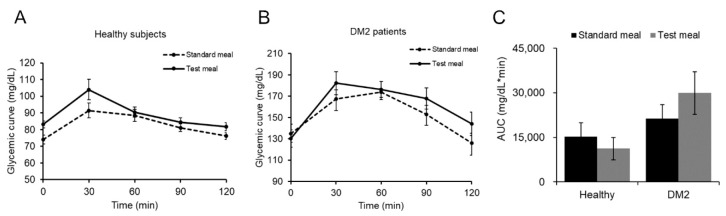
Postprandial glycemic curve of all individuals. Time course of changes in blood glucose levels after the standard or the test meal in (**A**) healthy subjects and (**B**) DM2 patients. Values are reported as the mean ± standard error (*n* = 10 per group). (**C**) Area under the curve of blood glucose concentration was measured between 0 and 120 min after the meals, T0-normalized values. Error bars represent standard error.

A paired analysis of the subjects showed that there was no significant difference in postprandial glucose elevation (*p* = 0.5, paired T-test) at various time points after the standard or the test meal, both in healthy subjects (Figure 5A) and in DM2 patients (Figure 5B).

The glycemic values shown in Table 5 indicate that the value of postprandial blood glucose was not significantly different in healthy subjects at 30 min after taking the standard or the test meal, i.e., the glycemic values were 91.6 ± 9.27 mg/dL and 104 ± 6.10 mg/dL, respectively. The same result was obtained in DM2 patients who showed no significant difference in blood glucose level at 30 min after taking the standard (167.3 ± 8.71 mg/dL) or the test (182.2 ± 10.78 mg/dL) meal. This result was maintained over time up to 120 min after meals. In fact, the glycemic curve of healthy subjects did not differ even at 120 min after the standard (76.1 ± 2.40 mg/mL) or the test (81.7 ± 2.35 mg/dL) meal, nor did that of diabetic patients (125.8 ± 9.23 mg/dL and 144.1 ± 11.09 mg/dL, respectively).

Similar results were obtained when the incremental area under the curve (iAUC) was compared between the two groups (Figure 5C). Therefore, these results prove that taking the test meal, added with *Annona cherimola* Miller, does not change the postprandial glycemia in diabetic subjects. The need to check the blood glucose level avoiding, in particular, the rise of postprandial blood glucose levels [54] makes the dietary intake of *Annona cherimola* Miller a useful strategy to manage the glucose response.

## 4. Conclusions

Nowadays, numerous health-promoting benefits are attributed to *Annona cherimola* Miller tropical fruit. In this work *Annona cherimola* Miller pulp extract showed interesting inhibitory abilities on the in vitro albumin glycation, one of the main causes underlying the diabetic complications onset. In addition, the extract was proven to be an effective competitive inhibitor of α-glucosidase, one of the main enzymes responsible for the inhibition of intestinal absorption of carbohydrates and of a lowered increase in postprandial glycemia. Although more studies and the enrollment of more healthy volunteers and DM2 patients into the clinical study are needed, our preliminary clinical results suggest that intake of *Annona cherimola* Miller pulp does not increase postprandial blood glucose levels, and therefore it could be conveniently consumed by diabetics in order to prevent chronic complications related to AGEs formation. A combination of the *Annona cherimola* Miller effects against oxidative stress, inflammation, chronic hyperglycemia, and protein glycation, which all are diabetes-related pathophysiological conditions, could make this fruit a good candidate from natural resources for the development of functional foods to be used in diabetes management and prevention of associated complications. In this regard, we believe that this work is in line with the current need to identify new anti-glycation agents to improve approaches to managing diabetic complications. Nevertheless, it is reasonable to consider that the *Annona cherimola* Miller beneficial properties could be explored for a pharmacological potential against other metabolic diseases. In conclusion, the relevance of *Annona cherimola* Miller fruit for diabetic patients management was further strengthened by results achieved with preliminary clinical study described in this work. In fact, diabetics or pre-diabetics, who are worried about their postprandial high glycemic values, can benefit from *Annona cherimola* Miller supplementation in their daily diet since the intake of this fruit does not cause a postprandial glycemic increase.

## Figures and Tables

**Table 1:** Nutritional values of standard meal.

	Cappuccino (120 g of Milk)	Brioche (45 g)
Proteins (g)	4	3.6
Fats (g)	4	9
Carbohydrates (g)	6	29
Calories (kcal)	77	205.5

The standard meal consisted of a cappuccino (120 g of milk) and a brioche (45 g).

**Table 2:** Nutritional values of edible portion (100 g) without seeds and peel of *Annona cherimola* Miller. Nutritional values were extracted from FoodData Central system of the USDA (United States Department of Agriculture) as a standard reference [39].

Nutrient	Unit	Amount
Water	g	79.66
Proteins	g	1.58
Lipids (total)	g	0.68
Sugars (total)	g	12.91
Carbohydrates	g	17.77
Fibers (total)	g	3
Energy	kcal	75
**Minerals**		
Calcium	mg	10
Iron	mg	0.27
Sodium	mg	7
Potassium	mg	288
Zinc	mg	0.16
Magnesium	mg	17
Phosphorus	mg	26
**Vitamins**		
Thiamine	mg	0.101
Vitamin C	mg	12.6
Niacin	mg	0.646
Riboflavin	mg	0.131
Folate	μg	23
Vitamin B-6	mg	0.258
Vitamin B-12	μg	0.000
Vitamin A	μg	0.000
Vitamin E	mg	0.27
Vitamin (IU)	IU	5
**Lipids**		
Fatty acids, total polyunsaturated	g	0.189
Fatty acids (total saturated)	g	0.234
Fatty acids (total monounsaturated)	g	0.055
Fatty acids, total trans	g	0.000
Cholesterol	mg	0.000

**Table 3:** Biochemical composition of *Annona cherimola* Miller pulp extract. All values are the average of three independent extractions, expressed as mean ± standard.

	TC	TP	Antioxidant Power	Radical Scavenging Activity
Method	Phenol-sulfuric acid	Folin–Ciocalteau	FRAP	DPPH
Reference	Glucose	Gallic acid	Ascorbic acid	Ascorbic acid
CE (mg/g) *	3306 ± 154.64	9.249 ± 0.41	1.134 ± 0.082	7.212 ± 0.034
CE (mg/mL) **	480.23 ± 22.46	1.343 ± 0.03	0.165 ± 0.012	1.047 ± 0.005

*: mg/g with respect to dry extract; **: mg/mL with respect to resuspended extract. TC: total carbohydrate; TP: total phenolic; FRAP: ferric reducing/antioxidant power; CE: cherimola pulp extract.

**Table 4:** Baseline characteristics of clinical study participants. All values are reported as the mean ± standard deviation.

	Healthy Subjects	DM2 Patients
Sex	N = 10 (F = 8; M = 2)	N = 10 (F = 7; M = 3)
Age (years)	29.3 ± 8.6	63.90 ± 9.35
Body weight (kg)	58.20 ± 10.34	65.12 ± 9.1
Height (cm)	163.4 ± 7.1	161.2 ± 4.3
Body Mass Index (kg/m^2^)	21.73 ± 2.62	25.06 ± 2.45
Fasting blood glucose (mg/dL)	74.22 ± 8.71	135.20 ± 28.91

DM2: type 2 diabetes mellitus. N: number of participants. F: female. M: male.

**Table 5:** Postprandial glycemic curve values of all subjects measured at different time points after the standard or the test meal. All values are reported in mg/dL. Data are reported as the mean ± standard error (*n* = 10 per group).

	Healthy Subjects		DM2 Patients	
Time (min)	Standard Meal	Test Meal	Standard Meal	Test Meal
0	74.0 ± 2.90	83.1 ± 2.08	134.9 ± 8.80	130.2 ± 8.21
30	91.6 ± 9.27	104 ± 6.10	167.3 ± 8.71	182.2 ± 10.78
60	88.5 ± 3.98	90.5 ± 2.95	173.2 ± 9.91	176.3 ± 7.27
90	81.1 ± 2.62	84.3 ± 2.79	152.8 ± 8.50	167.8 ± 10.08
120	76.1 ± 2.40	81.7 ± 2.35	125.8 ± 9.23	144.1 ± 11.09
